# A Massive Interstitial Cervical Leiomyoma Successfully Treated With Myomectomy: A Case Report

**DOI:** 10.1002/ccr3.71067

**Published:** 2025-10-01

**Authors:** Marah Mansour, Komait Swaid, Marah Razouk, Samaya Ibrahim Abdeen, Siba Baki, Mais Kwara, Ilda Kanbour

**Affiliations:** ^1^ Faculty of Medicine Tartous University Tartous Syrian Arab Republic; ^2^ Division of Colon and Rectal Surgery, Department of Surgery Mayo Clinic Rochester Minnesota USA; ^3^ Faculty of Medicine University of Aleppo Aleppo Syrian Arab Republic; ^4^ Faculty of Medicine Al Baath University Homs Syrian Arab Republic; ^5^ Al Bassel Hospital Homs Syrian Arab Republic; ^6^ Department of Pathology Damascus Hospital Damascus Syrian Arab Republic; ^7^ Department of Internal Medicine Aleppo University Hospital Aleppo Syrian Arab Republic; ^8^ Maternity University Hospital Damascus Syrian Arab Republic

**Keywords:** case report, cervical, fibroid, interstitial, leiomyoma, myomectomy

## Abstract

Cervical leiomyomas are rare pelvic tumors, with interstitial subtypes being the least common. This case highlights a 44‐year‐old female with a 14‐mm interstitial cervical leiomyoma, successfully treated with myomectomy. This approach preserved fertility, demonstrating its feasibility as an alternative to hysterectomy for select patients.

**Summary**

- Myomectomy is a viable fertility‐preserving option for interstitial cervical leiomyomas, even in challenging cases.
- This report emphasizes tailored surgical management based on tumor characteristics and patient preferences, avoiding the need for hysterectomy.

**Abbreviations**
AUB: abnormal uterine bleeding
CT: computed tomography
GnRH: gonadotropin‐releasing hormone
MRI: magnetic resonance imaging

## 1 Introduction

Leiomyomas, or fibroids, are the most common solid benign uterine tumors, affecting 20%–40% of women over 35. They arise from the clonal proliferation of myometrial cells and are hormonally responsive, primarily affecting reproductive‐age women. While 95% occur in the uterine corpus, cervical leiomyomas are rare (0.6%) [1, 2]. Up to 70% are asymptomatic, but symptomatic cases may present with abnormal uterine bleeding (AUB), hypermenorrhea, intermenstrual or postmenopausal vaginal bleeding, pelvic or lower back pain, dysmenorrhea, or bulk‐related symptoms like urine incontinence, stool constipation, sacral pain, dyspareunia, dyschezia, abdominal distension, and anemia, depending on size and location [1, 2, 3]. Ultrasonography is the first‐line imaging procedure for suspected leiomyoma; magnetic resonance imaging (MRI) and computed tomography (CT) are additional diagnostic tools [1, 3]. Excluding malignancy, particularly leiomyosarcoma, is critical [3]. Endocervical polyps and Nabothian cysts are benign differential diagnoses [4]. Asymptomatic fibroids do not require treatment [2]. Treatment is often surgical, requiring skilled management to address the risks of intraoperative hemorrhage and injuries to adjacent structures and to preserve fertility when needed [5]. Other options include conservative and interventional radiology approaches [1, 2]. Fibroids can complicate pregnancy, increasing risks of preterm labor, cesarean delivery, antepartum bleeding, fetal malpresentation, and growth restriction [1, 2]. We report a 20 cm cervical leiomyoma successfully managed by myomectomy.

## 2 Case History

A 44‐year‐old female patient, Gravida 5, Para 4, with a history of four vaginal deliveries (the last vaginal delivery was 13 years ago), presented with a 10‐year history of gradually increasing pelvic pressure and a sensation of pelvic heaviness. The patient also reported experiencing dyschezia (painful bowel movements) for the past 5 years and dyspareunia (painful intercourse) following her recent marriage. The patient denied significant menstrual bleeding. The patient is currently in the premenopausal stage. Her surgical history only includes ophthalmic surgery, with no medication or family history. Previous medical consultations indicated a high tumor likelihood and the potential requirement of a hysterectomy.

## 3 Differential Diagnosis, Investigations, and Treatment

**FIGURE 1:**
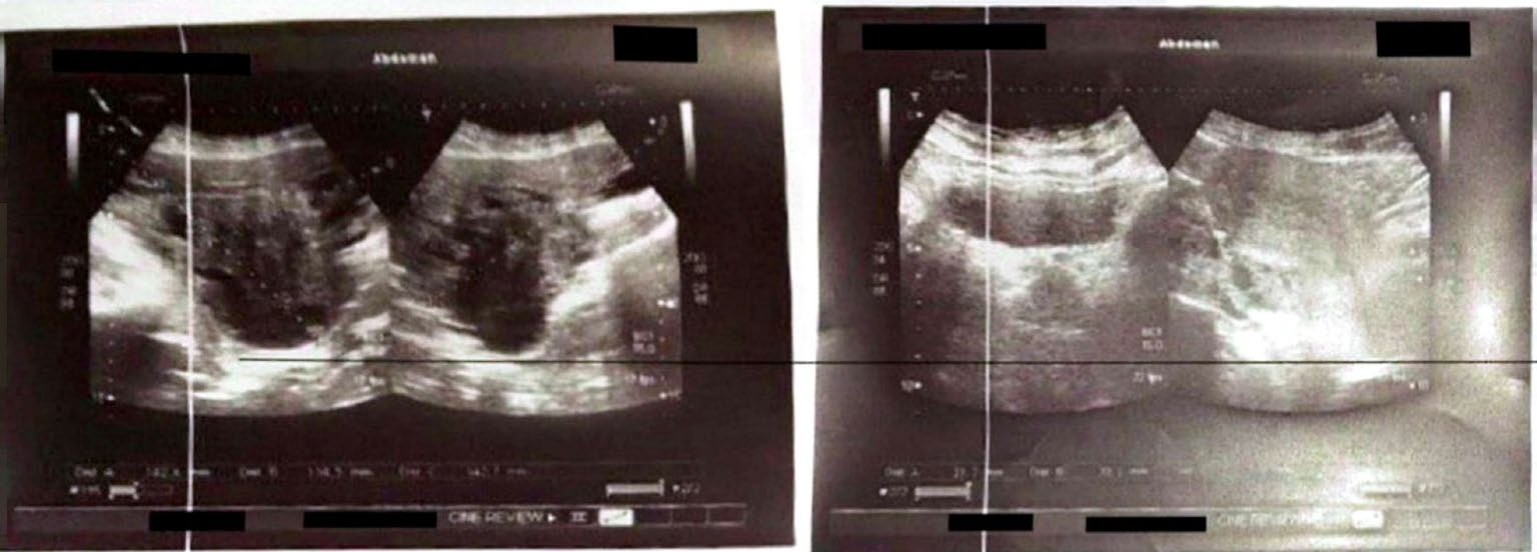
A Pelvic ultrasound shows cervix leiomyoma. The size of the mass is approximately 147 × 145 × 114.5 mm.

**FIGURE 2:**
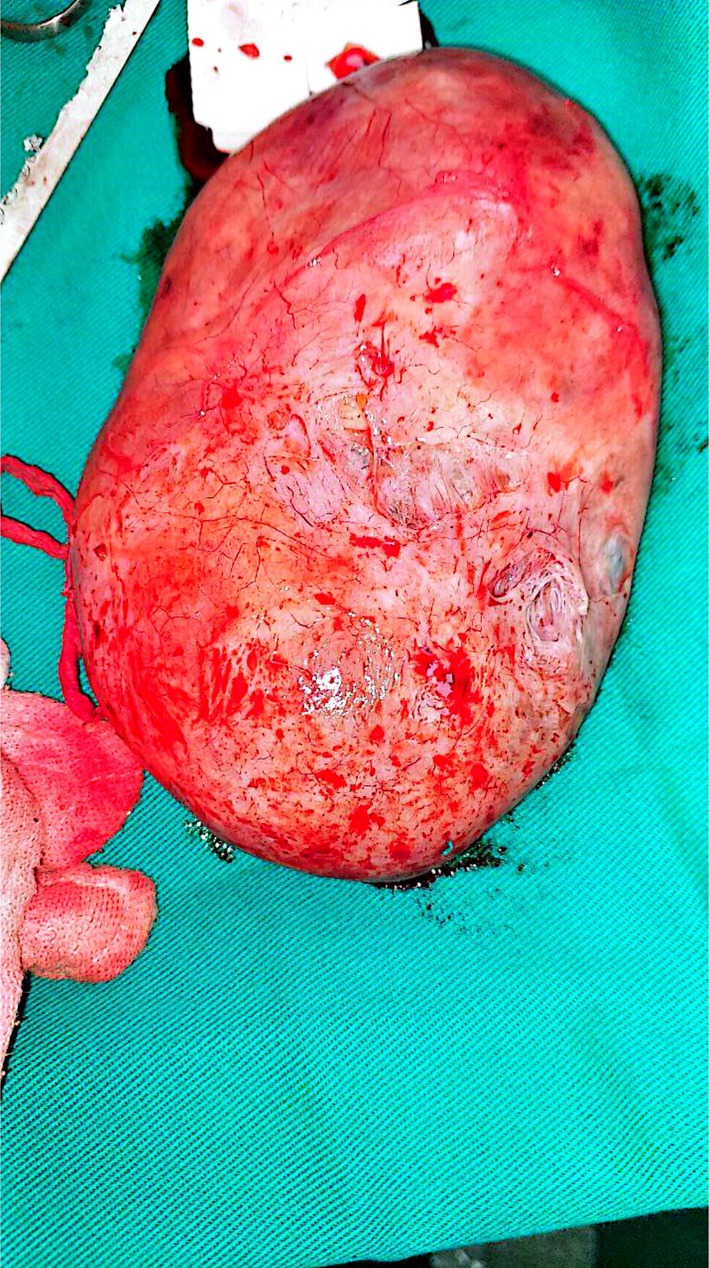
A mass of oval shape, characterized by clear boundaries, a hypoechoic appearance, heterogeneous internal structure, and the presence of fluid‐filled regions. The mass was identified on the left side of the pelvis, raising the possibility of a mixed ovarian cyst or neoplastic lesion.

**FIGURE 3:**
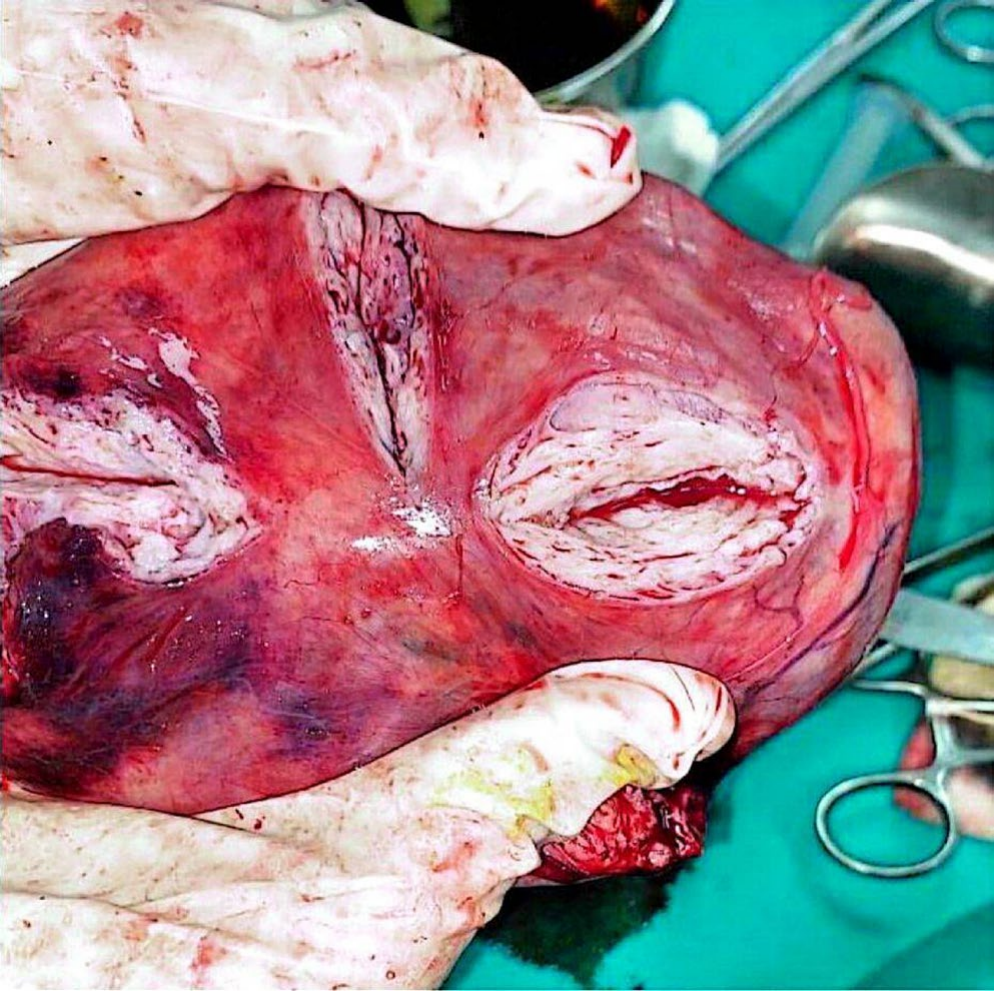
Postoperative image of the cervix pedunculated leiomyoma (fibroid) measures 16 × 11 × 5 cm. The cut surface reveals homogenous white tissue without necrosis or hemorrhage.

**FIGURE 4:**
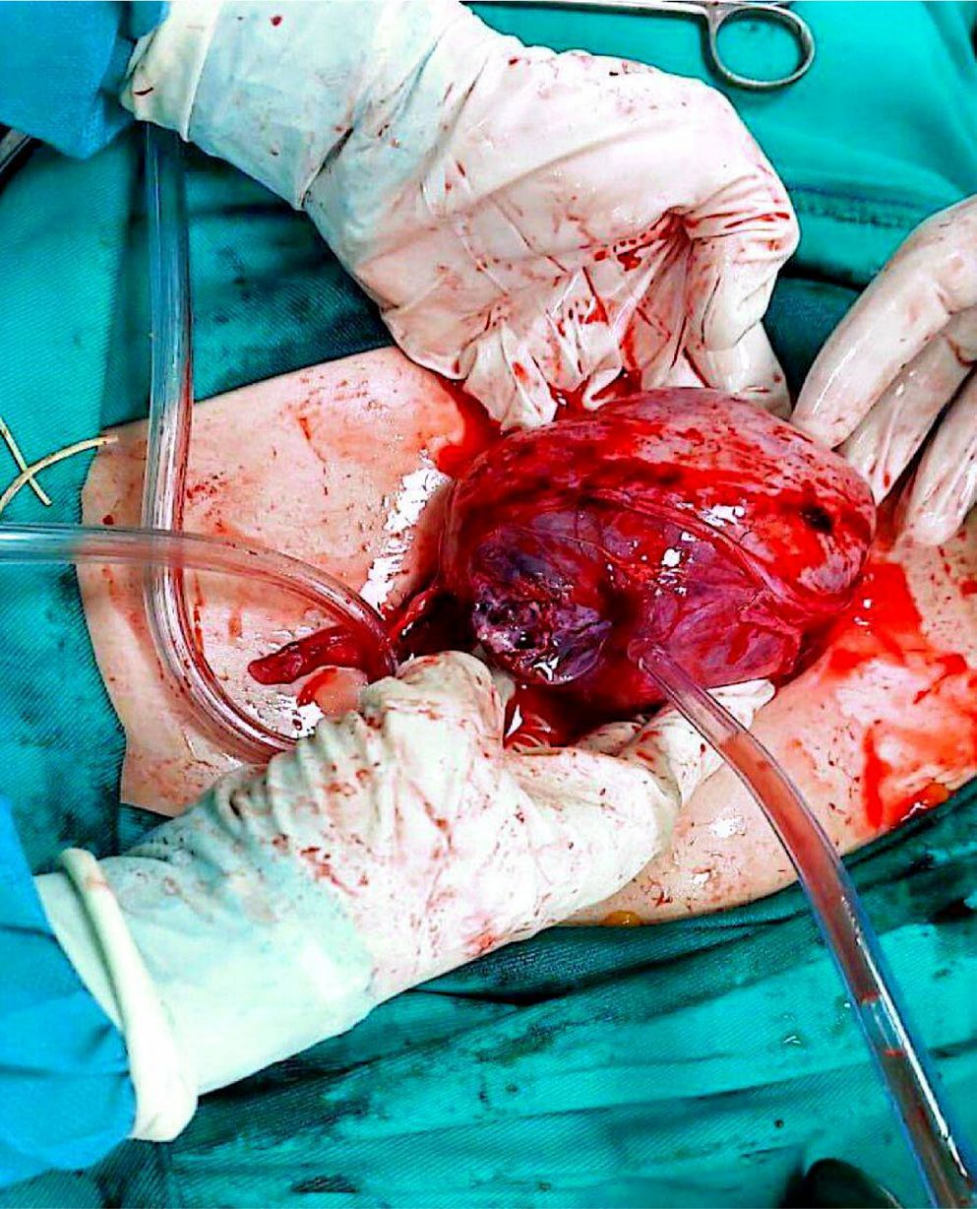
Intraoperative appearance of the large leiomyoma being suctioned/aspirated.

**FIGURE 5:**
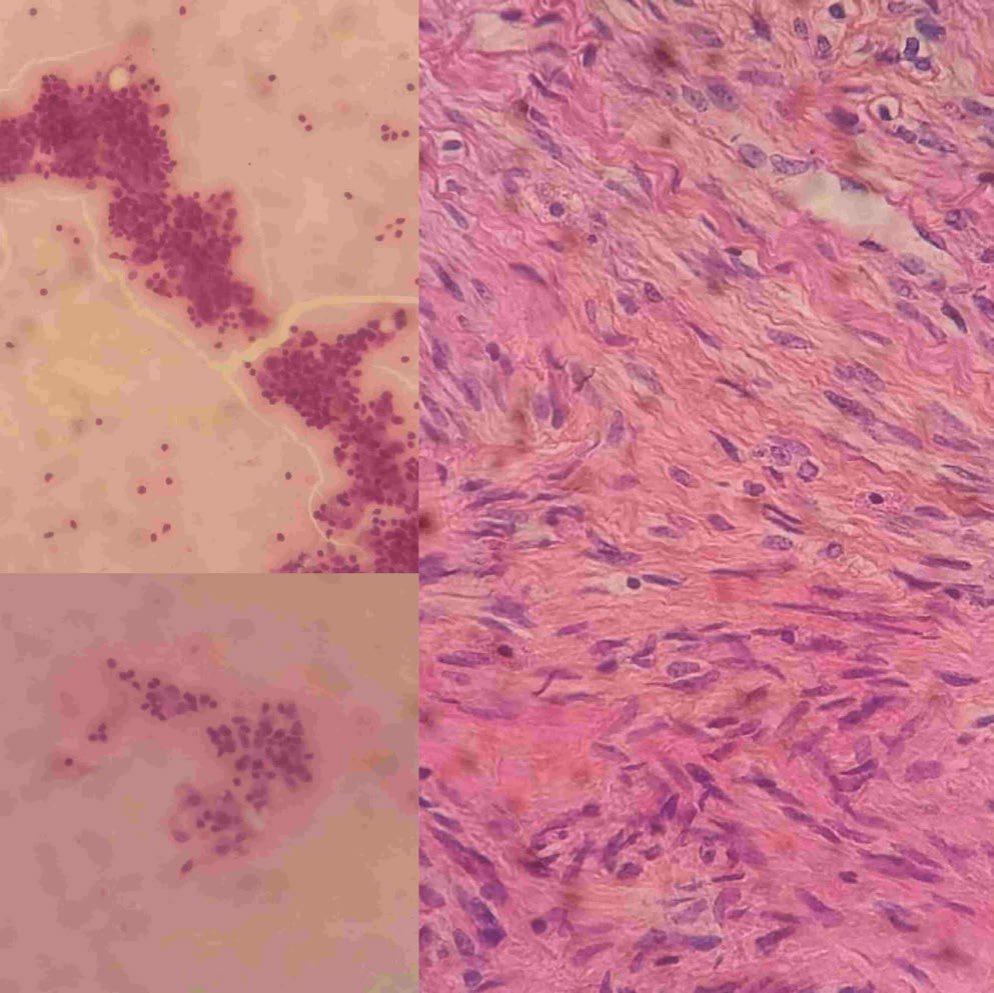
Histopathology, the proliferation of uniform, elongated, spindle‐shaped cells with a cigar‐shaped nucleus and the formation of bundles with different directions (whorled), with hyalinization, with few mitoses (less than 5 in HPF), consistent with leiomyoma, no evidence of malignancy.

On examination, a bimanual uterine examination revealed a palpable, non‐tender abdominal mass in the left iliac fossa, with indistinct inferior borders and restricted mobility. Laboratory investigations, including complete blood count, renal and liver function tests, and coagulation parameters, were unremarkable, except for a PTT value of 100 and a hemoglobin of 10.8 g/dL, consistent with mild anemia. A CA‐125 test was negative, ruling out ovarian malignancy. Abdominal ultrasound revealed an oval‐shaped mass with clear edges, a hypoechoic appearance, and heterogeneity, measuring (147 × 145 × 114.5 mm) (Figure 1). The mass's location on the left side of the pelvis, and further evaluation was recommended to ascertain the underlying etiology, with a differential diagnosis including mixed ovarian cyst or neoplasm. Despite the recommendations, the patient refused a total hysterectomy; therefore, we proceeded with only myomectomy to maintain the patient's fertility. Under general anesthesia, we performed the myomectomy. We discovered a large, well‐defined, bulky mass with macroscopic necrosis in the pelvis (Figures 2, 3, 4). The fibroid had grown at the expense of the cervix, with its center connected to the cervix and extending upwards toward the upper part of the uterus. Due to its large size, the mass required careful dissection. We aspirated approximately 400 mL of yellowish fluid from the mass, reducing its size, and then successfully removed it. We sent the sample for histopathological examination. Although macroscopic necrosis was present, it was deemed insignificant as malignancy was unlikely. Difficulties during the surgery included the fibroid extraction via Pfannenstiel incision (horizontal incision above the pubis), which was challenging due to the massive mass. Cytological and histological examinations revealed intersecting fascicles of uniform spindle cells with indistinct cell borders, eosinophilic cytoplasm, cigar‐shaped nuclei with tapered ends, and small nucleoli. No evidence of atypia, frequent mitoses, or necrosis was observed (hematoxylin–eosin stain, original magnification, ×60). Histopathology confirmed the diagnosis of a benign interstitial cervical leiomyoma with no evidence of malignancy (Figure 5).

## 4 Outcome and Follow‐Up

**FIGURE 6:**
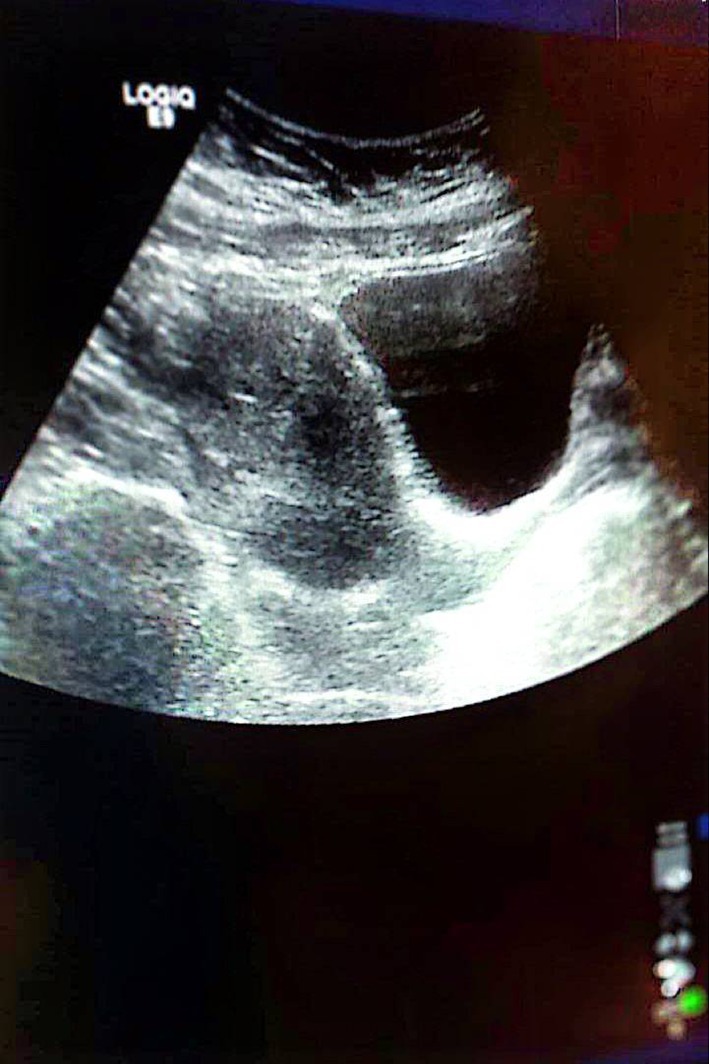
Two‐year follow‐up pelvic ultrasound performed with a half‐full bladder. The uterus is anteverted and positioned anteriorly. Uterine size and morphology are within normal limits. Both ovaries and adnexal structures appear normal, with no evidence of masses, cysts, or abnormal fluid collections.

The hospitalization period operation was uneventful. After we removed the suture, the patient's condition is stable, with regular menstruation resumed and no further complaints. Gonadotropin‐releasing hormone (GnRH) analogs were not used before surgery due to concerns about adverse effects. Postoperatively, the patient reported an unexpected increase in sexual desire, though the cause remains unclear. At the two‐year follow‐up, the patient remains asymptomatic with no recurrence of pelvic symptoms. A specialized pelvic ultrasound showed normal findings, with no evidence of mass recurrence (Figure 6). Routine laboratory investigations, including complete blood count, inflammatory markers, and renal function tests, were within normal limits. The medical team continues monitoring the patient regularly as part of long‐term surveillance.

## 5 Discussion

Uterine fibroids are the most common benign neoplasms affecting women, affecting 70% of women by menopause, originating from myometrial smooth muscle [3]. Cervical fibroids, a rare subtype (0.6% of cases), are classified into interstitial, supravaginal, and polypoid types [1, 3, 5, 6, 7, 8] because the cervix is primarily fibrous connective tissue with limited smooth muscle. Interstitial cervical leiomyomas, the rarest subtype as in our case, present with symptoms based on size, location, and the type of fibrosis [9], like chronic pelvic pain [1], dyspareunia, dyschezia, increasing abdominal distension, postmenopausal vaginal bleeding [1], vaginal discharge [1, 7, 10], vaginal bleeding [3, 5, 7, 8], urinary complaints [1, 7], mass extrusion in the vaginal orifice [5, 8, 10], heaviness, and mass in the abdomen [9]. The etiology includes estrogen dependency and histogenic theories such as misplaced embryonic progenitor cells going through lipoblastic differentiation, connective or smooth muscle tissue metaplastically changing into adipocytes, perivascular adipocytes, traumatized displacement of fat tissue, migration of pluripotent cells along uterine nerves and vessels, and fatty infiltration [3]. Diagnostic ultrasonography typically shows a circular, well‐defined, hypoechoic mass with heterogeneous features in cases of degeneration. Doppler ultrasound highlights relative hypovascularity [3]. A hyperechoic mass is typically linked to hemorrhage (cavernous, red degeneration) [4]. Heterogeneous structures are usually uterine fibroids [3]. Some studies reported hypoechoic cervix mass [5, 6, 7, 8, 10] and heterogeneous pelvis mass with evidence of internal vascularity [9]. Typical cervical leiomyoma tumor diameters reach up to 1.0 cm [3]. Our perimenopausal patient had completed childbearing and presented with pelvic pain and abdominal heaviness. Ultrasonography revealed an extensive (147 × 145 × 114.5 mm), hypoechoic, heterogeneous, and anechoic mass (Figure 1). Treatment options, whether medicinal, surgical, or minimally invasive, for symptomatic patients should be based on the size, quantity, and location of the fibroid [11]. Preoperative medical therapy, such as GnRH analogs, can reduce fibroid size and vascularity three months before surgery [1, 6]. Our patient refused medical treatment due to concerns about adverse effects. Interventional radiology techniques, including uterine artery embolization, have shown limited but promising outcomes as adjuncts or alternatives to surgery, particularly in cases unsuitable for extensive surgical intervention [1]. Surgical treatment presents unique challenges due to their pelvic location and the associated anatomical distortions [1]. The upward and outward displacement of uterine vessels, elevation of the bladder, and deformation of the ureters increase the complexity of surgical procedures [6]. These factors significantly influence the duration and technical demands of minimally invasive approaches, necessitating advanced surgical expertise [1]. Hysterectomy is the standard treatment for symptomatic leiomyomas; however, fertility preservation remains a priority for patients who desire childbearing. In our case, the patient declined a hysterectomy, and a myomectomy was performed, consistent with individualized care for preserving reproductive potential [1]. In our case, the size and characteristics of the leiomyoma compounded the difficulties. The mass required an incision and 400 mL of fluid aspiration to facilitate extraction. The fibrous stalk was completely excised, demonstrating a successful myomectomy (Figure 4). Postoperative recovery was uneventful, and histopathology confirmed the diagnosis of cervical leiomyoma. The complications include bladder or urethral compression, intermenstrual bleeding, pelvic pain, degenerative phenomena, prolapse with infection, and torsion [7]. The rarity of massive cervical fibroid polyps adds to the diagnostic and therapeutic complexity. In our case, despite the mass's significant size, there were no intraoperative or postoperative complications, emphasizing the importance of fertility‐preserving surgery planning.

## 6 Conclusion

Massive cervical leiomyomas are rare, predominantly affecting reproductive‐age women. Management options include a hysterectomy or myomectomy, guided by the patient's age and fertility goals. Surgical intervention, often complex due to bleeding risks, necessitates expertise and advanced hemostatic techniques to ensure optimal outcomes.

## Author Contributions

**Marah Mansour:** project administration, supervision, validation, writing – review and editing. **Komait Swaid:** supervision, writing – review and editing. **Marah Razouk:** writing – original draft. **Samaya Ibrahim Abdeen:** investigation, writing – original draft. **Siba Baki:** writing – original draft. **Mais Kwara:** writing – original draft. **Ilda Kanbour:** data curation, investigation, resources.

## Ethics Statement

The authors have nothing to report.

## Consent

Written informed consent was obtained from the patient for publishing this case report and any accompanying images. A copy of the written consent is available for review by the Editor‐in‐Chief of this journal on request.

## Conflicts of Interest

The authors declare no conflicts of interest.

## Data Availability

The authors have nothing to report. All data (of the patient) generated during this study is included in this published article.
